# Maternal mortality following caesarean section in a low-resource setting: a National Malawian Surveillance Study

**DOI:** 10.1136/bmjgh-2024-016999

**Published:** 2024-11-24

**Authors:** Jennifer Riches, Yamikani Chimwaza, Bertha Immaculate Magreta Chakhame, Jack Milln, Hussein H Twabi, Rosemary Bilesi, Luis Gadama, Fannie Kachale, Annie Kuyere, Lumbani Makhaza, Regina Makuluni, Laura Munthali, Owen Musopole, Chifundo Ndamala, Deborah A Phiri, Louise Afran, Amie Wilson, Shakila Thangaratinam, Abi Merriel, Catriona Waitt, Maria Lisa Odland, James Jafali, David Lissauer

**Affiliations:** 1 Department of Women’s and Children’s Health, University of Liverpool, Liverpool, UK; 2 Malawi-Liverpool-Wellcome Clinical Research Programme, Blantyre, Malawi; 3 Neonatal Health, University of Malawi Kamuzu College of Nursing, Lilongwe, Central, Malawi; 4 Reproductive Health Directorate, Government of Malawi Ministry of Health, Lilongwe, Central Region, Malawi; 5 Kamuzu University of Health Sciences, Blantyre, Malawi; 6 Liverpool School of Tropical Medicine, Liverpool, UK; 7 Liverpool Women’s Hospital NHS Foundation Trust, Liverpool, UK; 8 Department of Pharmacology and Therapeutics, Institute of Systems, Molecular & Integrative Biology, University of Liverpool, Liverpool, UK; 9 Infectious Diseases Institute, Makerere University, Kampala, Uganda; 10 Department of Public Health and Nursing, Norwegian University of Science and Technology, Trondheim, Norway

**Keywords:** Maternal health, Surgery, Infections, diseases, disorders, injuries, Global Health, Health systems

## Abstract

**Background:**

Caesarean section (CS) is the most common major surgery conducted globally, with rates rising. CS also contributes to maternal morbidity and mortality, with increased risks in low-resource settings. We conducted a detailed review of maternal deaths from 2020 to 2022 in Malawi to determine the burden of deaths related to CS, avoidable health system factors, and causes of death associated with this procedure.

**Methods:**

Data were collected regarding every maternal death occurring across all district and central hospitals in Malawi, alongside facility-level aggregated birth data. Maternal deaths were reviewed by facility-based multidisciplinary teams with subsequent confirmation of cause of death by obstetricians according to international criteria. Logistic regression was applied to estimate the odds of associations of leading causes of death with CS while adjusting for potential confounders.

**Results:**

Despite a low national CS rate, most deaths occurred following CS (51.8%, 276/533). Women who delivered by CS were five times (OR 5.60, 95% CI 4.74 to 6.67) more likely to die than women who delivered vaginally. The leading causes of death following CS were postpartum haemorrhage (26.0%, 68/277), eclampsia (15.6%, 41/277) and infection (14.1%, 37/277). Deaths from pregnancy-related infection were more often associated with CS (OR 2.03, 95% CI 1.12 to 3.72). Health system factors more frequently associated with deaths following CS than vaginal birth included ‘prolonged abnormal observations without action’ (p=0.006), ‘delay in starting treatment’ (p=0.006) and ‘lack of blood transfusion’ (p=0.03).

**Conclusions:**

We found a high burden of maternal death following CS in this low-resource setting. Until now, international attention and many clinical trials have been focused on improving the safety of vaginal birth. Our findings highlight the need to ensure the safe and appropriate use of this potentially life-saving intervention to reduce maternal deaths. To avoid the high burden of death following CS we highlight, there is urgent need to develop and trial CS-specific interventions.

**WHAT IS ALREADY KNOWN ON THIS TOPIC**

**WHAT THIS STUDY ADDS**

- This study confirms a high burden of death following CS compared with following vaginal birth in a low-resource African setting. The leading medical causes of death following CS were haemorrhage, eclampsia and infection. Deaths from pregnancy-related infections were more common among women who delivered by CS.

**HOW THIS STUDY MIGHT AFFECT RESEARCH, PRACTICE OR POLICY**

- Multiple remediable health systems factors were identified as contributing to CS-related deaths. There is an urgent need to develop and trial interventions to improve the safety of CS in low-resource settings.

## Background

 Caesarean section (CS) is the most frequently performed surgical procedure worldwide.^1^ Rates of CS birth are rising across all global regions.^1^ Latest estimates reveal that 21.1% of women worldwide gave birth by CS between 2010 and 2018,^1^ expected to rise to 28.5% by 2030.^1^ CS rates remain a topic of global controversy (‘too many too soon’ vs ‘too little too late’)^2^,^4^ as both overuse and underuse of the procedure may contribute to complications.^5^ However, in certain situations, CS is (and will remain) necessary to achieve optimal outcomes in both maternal and neonatal health.^6^ As such, access to safe CS is a key component of the comprehensive emergency obstetric care package recommended by the WHO,^6^ and an important focus for any health system wishing to improve maternal health.

Although many low-income and middle-income countries (LMICs) remain underserved by this potentially life-saving surgical procedure,^7^ it is in low-resource contexts that CS is most frequently associated with a significant risk of maternal morbidity and mortality.^8^ In view of this, we conducted this detailed national review of postnatal maternal deaths in Malawi, a low-income country in the WHO African Region, with a focus on deaths following CS. We sought to determine the burden of deaths following CS, the health system factors associated with these deaths, and the medical causes of death among women undergoing CS.

## Methods

### Study setting

Data for this study were collected from 33 healthcare facilities across Malawi, including all 4 central (tertiary level) hospitals, all 27 district hospitals (online supplemental figure S1), and 2 health centres. Deaths occurring outside of these facilities (eg, in the community and in private for-profit and private mission hospitals) are reported by the corresponding district hospital and are, therefore, also included in our analysis.

### Study design and participants

We conducted a retrospective analysis of individual-level maternal mortality data prospectively collected using a digital surveillance platform. The observation period was between 1 August 2020 and 31 December 2022.

Participants eligible for enrolment in the study included all women who died from ‘a cause related to or aggravated by pregnancy or its management or within 42 days of the end of the pregnancy’, in keeping with the WHO definition of maternal death.^9^ Maternal deaths were included if they had been audited by a local maternal death surveillance and response (MDSR) committee to provide information regarding the cause and circumstances of the death. Deaths which were reported but not audited were excluded due to lack of sufficient detail. For this analysis, we limited the study population to those who died postnatally and for whom a mode of delivery was documented (online supplemental figure S2). The early postnatal period was defined as the first 24 hours after birth, and the late postnatal period from 24 hours to 42 days after birth.

### Data collection

Data were collected using a digital maternal health surveillance platform (MATSurvey), established by the Malawi-Liverpool-Wellcome Research Programme and the Ministry of Health (MOH) of Malawi in 2020 to digitalise and enhance the surveillance potential of maternal health data (online supplemental figure S3).^10^ Case narratives describing each maternal death were compiled by MDSR committees. Aggregate data from facilities, including information about the weekly number and mode of births, were used to calculate an average CS rate. Data were uploaded to the MATSurvey platform using mobile data collection tools loaded onto tablets (OpenDataKit V.1.21.0). This task was performed by ‘Safe Motherhood Coordinators’ at each study site; nurse-midwives trained by the MOH in case-finding and data collection, responsible for routine data collection from clinical notes, handover files and hospital registers as well as the completion of MOH audit/review proformas for each maternal death during local MDSR review of the death. Data used in this analysis were fully anonymised and were available to the authors through permission from the MOH of Malawi and the College of Medicine Research Ethics Committee.

### Determining cause of death and avoidable health system factors

The cause of each maternal death was initially determined by local MDSR committees. The case narrative recorded for each death was then independently confirmed by an obstetrician, who mapped the cause of death to the WHO International Classification of Diseases categories,^9^ adapted for use in Malawi by the MOH. The cause of death was defined using WHO principles as ‘the disease or condition that initiated the morbid chain of events leading to the death’.^9^ Where discrepancies occurred, a second obstetric opinion was sought. To attribute avoidable factors to each death, local MDSR committees selected factors from a standardised list provided by the MOH. These included ‘healthcare worker factors’ (related to healthcare worker practices such as monitoring, referral and timeliness of action), ‘administrative factors’ (related to resources, infrastructure, transport and communication), ‘patient/family factors’ (related to health-seeking behaviour, barriers to care) and ‘traditional birth attendant/community factors’. Each case could be ascribed to an unlimited number of avoidable factors.

### Data analysis

Categorical variables were summarised using frequencies and proportions, and continuous variables using medians and IQRs. All modes of delivery were summarised as proportions of the total number of women who died after giving birth (ie, spontaneous vaginal delivery, vacuum-assisted delivery, breech, destructive procedures and CS). These were then grouped into vaginal deliveries (including spontaneous, vacuum, breech and destructive) and caesarean deliveries for further analysis. Demographic and clinical characteristics of each group and avoidable health system factors associated with deaths were described and compared using frequencies/proportions (with 95% CIs) and χ^2^ tests for significance in differences between the groups. Causes of death were then summarised for the caesarean group and the vaginal delivery group and compared in the same way.

To further explore the relationship between mode of delivery and risk of death, logistic regression analysis was performed. Aggregated data from the 33 participating facilities were used to provide denominators for the calculation of an OR for the risk of death following CS compared with the risk of death following vaginal birth. Next, regression analysis was used to compare causes of death between women who died following CS and women who died following vaginal birth. Death following CS rather than following vaginal delivery was treated as the outcome of interest, and leading causes of death were included in the model as ‘exposures’ to calculate the odds of each cause of death for women undergoing CS compared with those who delivered vaginally. The model was adjusted for demographic and clinical factors found to be significant in stepwise regression (educational level and timing of death).

### Definition of variables

‘Vaginal delivery’ includes spontaneous vaginal deliveries, assisted vaginal deliveries (forceps and vacuum), breech vaginal deliveries and destructive procedures. ‘CS rate’ was defined using the WHO definition as the number of women delivering by CS as a percentage of the total number of live births.^1^ ‘Stable’ condition and ‘critical’ condition were determined subjectively by healthcare staff at facilities based on the patient’s vital signs and overall presentation. Infectious causes of death were then subdivided into ‘pregnancy-specific’ (infection directly related to pregnancy or genital tract infections) and ‘non-pregnancy-specific’ infection (such as malaria or tuberculosis).

### Patient and public involvement

At the inception and implementation of the MATSurvey digital platform, information was presented to the patient and public involvement group who advise our research group and their feedback was sought. Findings from this analysis (as well as broader analysis of maternal deaths during this period) have been presented to community and public stakeholders in partnership with the MOH of Malawi.

## Results

In total, 1162 maternal deaths were reported by facilities during the study period; 809 deaths were audited. Of these deaths, 533 occurred postnatally with a recorded mode of delivery and were therefore suitable for inclusion in our sample (online supplemental figure S2). 276 women who died postnatally died following a CS (51.8% (95% CI 47.4% to 56.1%)), and 257 women died following a vaginal birth (48.2% (95% CI 43.9% to 52.6%)). Of vaginal births, 240 (93.4%) were spontaneous vaginal deliveries, 10 vacuum-assisted vaginal deliveries (3.9%), 4 breech vaginal deliveries (1.6%) and 3 destructive procedures (1.2%).

### Risk of death following CS compared with risk of death following vaginal birth

Overall, 89 098 CS and 465 375 vaginal births were recorded during the study period. The mean CS rate for participating facilities was 16.7% of live births. The mortality rate for women undergoing CS was 3.1 per 1000 CS. Based on the proportion of deaths which occurred after CS in our sample, women who delivered by CS had a risk of death more than five times that of women who delivered vaginally (OR 5.60, 95% CI 4.74 to 6.67, p<0.001).

### Clinical and demographic characteristics of women who died by mode of delivery

The demographic and clinical characteristics of women who died in the postnatal period are shown in table 1. Women who died following CS had more antenatal attendances (4 vs 3, p=0.018) and were of more advanced gestation (38 vs 37 weeks, p=0.011) than women who died following a vaginal birth. Women who died following a CS had higher levels of education (25.0% vs 14.5% with secondary/tertiary education, p=0.016), their deaths more frequently occurred in the early postnatal period (60.9% vs 49.0%, p=0.019), (defined as the first 24 hours after birth) and they were more often stable on arrival to the facility where they died (52.5% vs 33.5%, p=<0.001) than women who delivered vaginally (table 1). ORs for maternal deaths following CS by demographic and clinical exposures are presented in online supplemental table S1.

**Table 1:** Demographic characteristics, clinical characteristics, health system factors and causes of death of women who died postnatally

Characteristics	CS (n=276)		Vaginal birth (n=257)		P value
	Median	IQR	Median	IQR	
Age (years)	28	(22, 34)	27	(21, 34)	0.422
Parity (births)	2	(1, 4)	3	(1, 4)	0.710
ANC (appointments)	4	(3, 5)	3	(2, 4)	**0.018**
Gestation (weeks)	38	(36, 38)	37	(34, 38)	**0.011**

	n	Proportion (95% CI)	n	Proportion (95% CI)	
Age groups					
<20 years	43	15.6% (11.6 to 20.5%)	42	16.3% (12.2 to 21.6%)	0.765
20–25 years	71	25.7% (20.8 to 31.4%)	75	29.2% (23.8 to 35.2%)	
26–35 years	99	35.9% (30.3 to 41.9%)	83	32.3% (26.7 to 38.4%)	
>35 years	63	22.8% (18.1 to 28.3%)	57	22.2% (17.4 to 27.9%)	
Marital status					
Married	235	92.5% (88.4 to 95.3%)	210	91.7% (87.2 to 94.8%)	0.946
Single	14	5.5% (3.2 to 9.3%)	14	6.1% (3.5 to 10.3%)	
Other	5	2.0% (0.7 to 4.8%)	5	2.2% (0.8 to 5.3%)	
Educational level					
None	70	27.8% (22.4 to 33.8%)	79	34.6% (28.6 to 41.3%)	**0.016**
Primary	119	47.2% (41.0 to 53.6%)	116	50.9% (44.2 to 57.5%)	
Secondary	46	18.3% (13.8 to 23.7%)	28	12.3% (8.5 to 17.4%)	
Tertiary	17	6.7% (4.1 to 10.8%)	5	2.2% (0.8 to 5.3%)	
Parity					
0	9	4.5% (2.2 to 8.6%)	10	5.0% (2.5 to 9.2%)	0.547
1	60	29.9% (23.7 to 36.8%)	47	23.4% (17.8 to 30.0%)	
2	34	16.9% (12.2 to 23.0%)	36	17.9% (13.0 to 24.1%)	
3	37	18.4% (13.4 to 24.6%)	34	16.9% (12.2 to 23.0%)	
>3	61	30.3% (24.2 to 37.3%)	74	36.8% (30.2 to 43.95)	
Timing of death					
Early postnatal*	157	60.9% (54.6 to 66.8%)	119	49.0% (42.5 to 55.4%)	**0.019**
Late postnatal†	100	38.8% (32.8 to 45.0%)	124	51.0% (44.6 to 57.5%)	
Postnatal (timing unknown)	19	7.4% (4.6 to 11.4%)	14	5.8% (3.3 to 9.7%)	
Gestation at death					
<28 weeks	4	1.6% (0.5 to 4.2%)	11	4.7% (2.5 to 8.5%)	0.176
28–31 weeks	21	8.2% (5.2 to 12.4%)	22	9.4% (6.1 to 14.1%)	
32–36 weeks	55	21.4% (16.7 to 27.0%)	53	22.7% (17.6 to 28.8%)	
37–42 weeks	177	68.9% (62.8 to 74.4%)	147	63.1% (56.5 to 69.2%)	
HIV status					
HIV positive	22	9.3% (6.1 to 14.0%)	30	14.7% (10.3 to 20.5%)	0.081
HIV negative	214	90.7% (86.0 to 93.9%)	174	85.3% (79.5 to 89.7%)	
HIV taking ART					
Not taking ART	10	45.5% (25.1 to 67.3%)	13	43.3% (26.0 to 62.3%)	0.879
Taking ART	12	54.5% (32.7 to 74.9%)	17	56.7% (37.7 to 74.0%)	
ANC					
No ANC	8	3.4% (1.6 to 6.8%)	21	10% (6.4 to 15.1%)	**0.004**
Received ANC	230	96.6% (93.2 to 98.4%)	189	90% (84.9 to 93.6%)	
Admitted from					
Another facility	173	62.7% (56.7 to 68.3%)	155	60.3% (54.0 to 66.3%)	0.574
Home/community	103	37.3% (31.7 to 43.3%)	102	39.7% (33.7 to 46.0%)	
Condition at admission					
Critically ill	128	46.4% (40.4 to 52.4%)	151	58.8% (52.5 to 64.8%)	**<0.001**
Dead on arrival	3	10.9% (2.8 to 3.4%)	20	7.8% (4.9 to 11.9%)	
Stable	145	52.5% (46.5% to 58.5%)	86	33.5% (27.8 to 39.6%)	
Health system factors					
Any healthcare worker factor	246	89.1% (84.7 to 92.4%)	221	86.0% (81.0 to 89.9%)	0.272
Any administrative factor	141	51.1% (45.0 to 57.1%)	130	50.6% (44.3 to 56.8%)	0.908
Any patient/family factor	100	36.2% (30.6 to 42.2%)	142	55.3% (48.9 to 61.4%)	**<0.001**
Any TBA/community factor	16	5.8% (3.5 to 9.4%)	27	10.5% (7.2 to 15.1%)	**0.046**

p values in bold highlight statistically siginificant different between groups.

* Early postnatal=first 24 hours after birth.

† Late postnatal =24hours to 42 days after birth.

ANC, Antenatal care; ART, Anti-retroviral therapy; CS, caesarean section; IQR, Interquartile range; TBA, traditional birth attendant.

### Avoidable health system factors by mode of delivery

The frequency with which health system factors were associated with maternal deaths is shown in table 1. ‘Healthcare worker’ factors were found to be present in 89.1% of deaths following CS compared with 86.0% of deaths following vaginal birth (p=0.272). ‘Administrative’ factors were found to be present in 51.1% of deaths following CS, and 50.6% of deaths following vaginal birth (p=0.908). ‘Patient and family’ factors were more common in cases of maternal death which followed vaginal birth (55.3% vs 36.2%, p<0.001), as were ‘traditional birth attendant/community’ factors (10.5% vs 5.8%, p=0.046).

**Figure 1:**
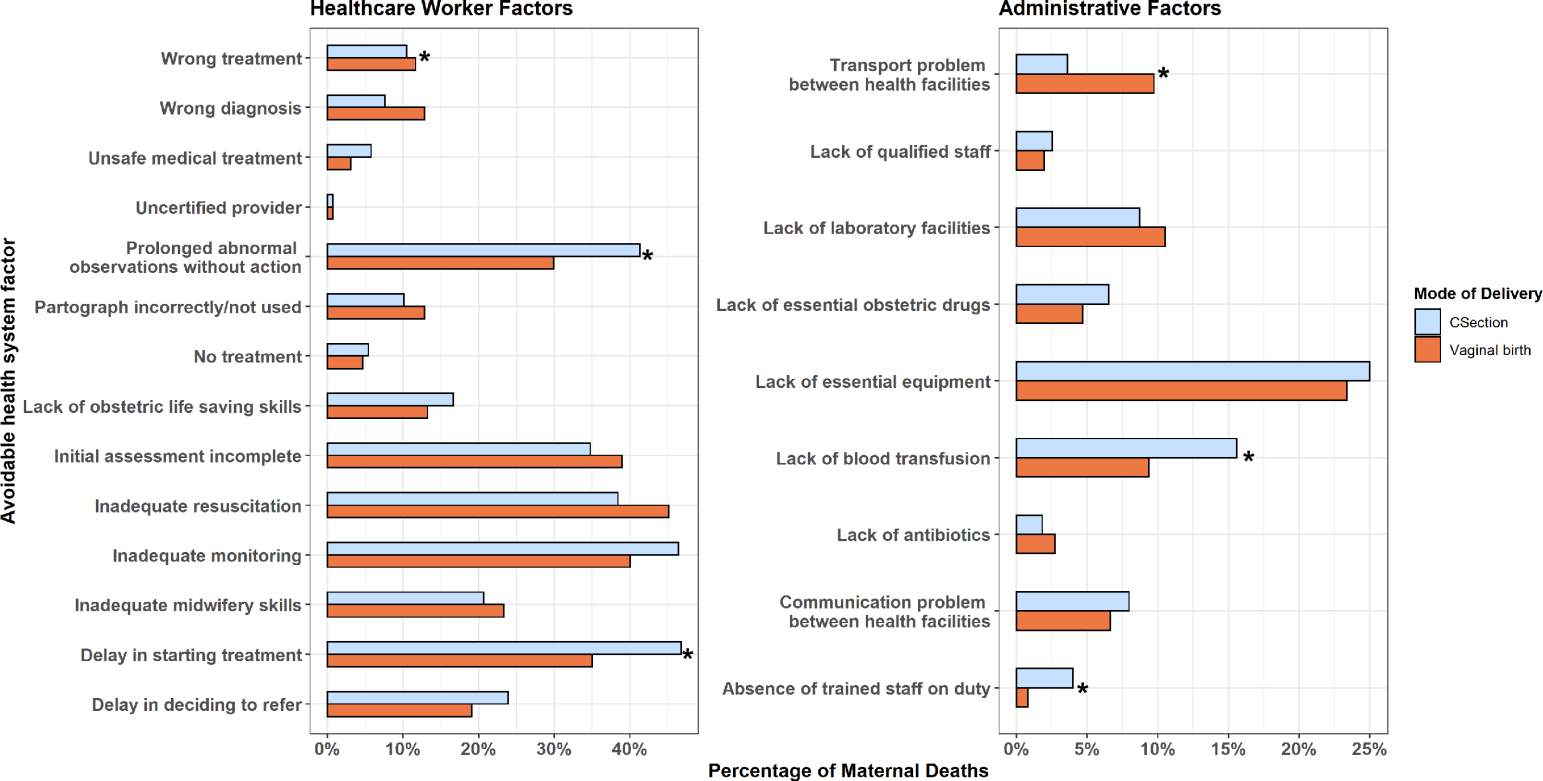
Avoidable factors involved in maternal deaths by mode of delivery. *Significant difference between groups.

Among the subcategories of healthcare worker and administrative factors, ‘prolonged abnormal observations without action’, ‘inadequate monitoring’, ‘delay in starting treatment’, ‘lack of essential equipment’ and ‘lack of blood transfusion’ were most frequently linked to deaths following CS (figure 1). Factors more frequently associated with a particular mode of delivery are highlighted in figure 1 and online supplemental table S2. ‘Prolonged abnormal observations without action’, ‘delay in starting treatment’ (46.7% vs 35.0%, p=0.006), ‘lack of blood transfusion’ (15.6% vs 9.3%, p=0.030) and ‘absence of trained staff on duty’ (4.0% vs 0.8%, p=0.016) were more frequently associated with CS than with vaginal birth.

### Cause of maternal death by mode of delivery

Cause of death was determined for 504 women who died postnatally; for 29 women, there was insufficient information available to determine causation (14 CS, 15 vaginal). Cause of death by mode of delivery is displayed in table 2 and online supplemental table S3. The leading causes of death among those giving birth by CS were postpartum haemorrhage (n=68 (26.0%)), followed by eclampsia (n=41 (15.6%)) and pregnancy-specific infection (n=37 (14.1%)). These were also the leading causes of death among those women who delivered vaginally.

**Table 2:** Causes of maternal death by mode of delivery

Cause of death	CS (n=262)		Vaginal birth (n=242)	
	Proportion (95% CI)		Proportion (95% CI)	
Postpartum haemorrhage	68	26.0% (20.8 to 31.8%)	93	38.4% (32.3 to 44.9%)
Eclampsia	41	15.6% (11.6 to 20.7%)	28	11.6% (8.0 to 16.4%)
Pregnancy-specific infection*	37	14.1% (10.3 to 19.1%)	28	11.6% (8.0 to 16.4%)
Ruptured uterus	31	11.8% (8.3 to 16.5%)	9	3.7% (1.8 to 7.1%)
Antepartum haemorrhage	26	9.9% (6.7 to 14.4%)	1	0.4% (0.02 to 2.6%)
Pre-eclampsia	22	8.3% (5.5 to 12.6%)	19	7.8% (4.9 to 12.2%)
Non-pregnancy specific infection†	15	5.7% (3.4 to 9.5%)	42	17.4% (12.9 to 22.9%)
Complications of anaesthesia	8	3.1% (1.4 to 6.2%)	0	–
Medical complications in pregnancy‡	8	3.1% (1.4 to 6.2%)	17	7.0% (4.3 to 11.2%)
Peripartum cardiomyopathy	5	1.9% (0.7 to 4.6%)	4	1.7% (0.5 to 4.5%)

* Pregnancy-specific infection refers to infections directly related to pregnancy such as chorioamnionitis or endometritis.

† Non-pregnancy-specific infection refers to infections not directly related to pregnancy such as malaria or tuberculosis.

‡ Includes venous complications, diabetes, cardiovascular disease, gastrointestinal conditions, central nervous system conditions, cancer, anaemia, respiratory conditions and haematological conditions.

**Figure 2:**
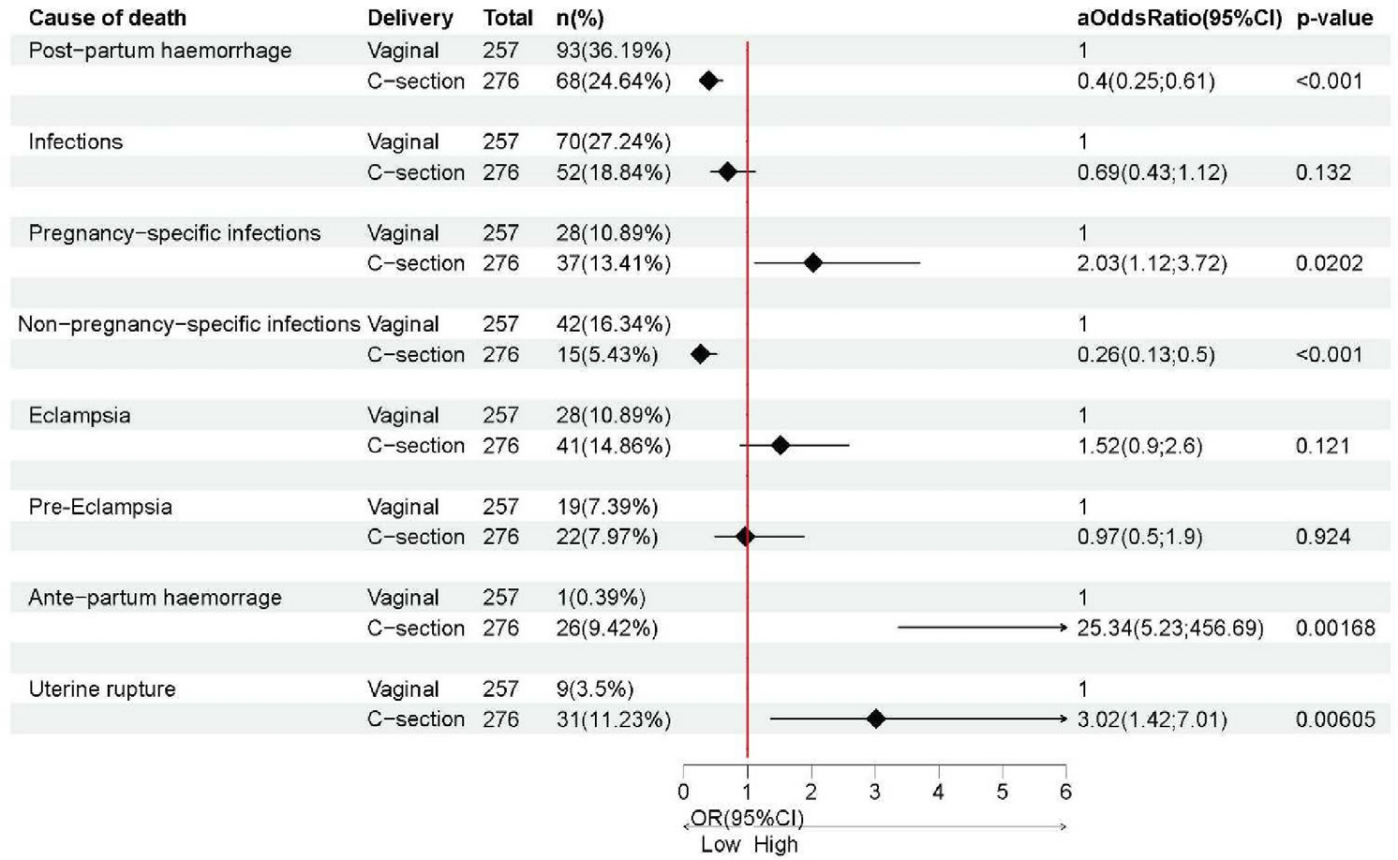
Forest plot showing odds (95% Cl) of association between each cause of death and mode of delivery.

Figure 2 shows results from multiple logistic regression, comparing the odds of dying from each main cause of death (against other causes of death) between CS and vaginal deliveries while adjusting for potential confounders (educational level and timing of death). Briefly, death following CS was more frequently associated with death from pregnancy-specific infections (OR 2.03, 95% CI 1.12 to 3.72) and antepartum haemorrhage (OR 25.3, 95% CI 5.23 to 456.7); but less frequently associated with postpartum haemorrhage (OR 0.4, 95% CI 0.25 to 0.61) and non-pregnancy specific infection (OR 0.26, 95% CI 0.13 to 0.5). Unadjusted ORs are presented in online supplemental table S4.

## Discussion

We used data from secondary and tertiary hospital facilities in Malawi to conduct a comprehensive review of all postnatal maternal deaths occurring nationally between 2020 and 2022. Women who gave birth by CS were over five times more likely to die than women who delivered vaginally, and over half of all postnatal maternal deaths followed a CS. Leading causes of death for women who delivered by CS were postpartum haemorrhage, eclampsia and infection. We found several modifiable health system factors to be more frequently associated with CS including ‘“prolonged abnormal observations without action’, ‘delay in starting treatment’ and ‘lack of blood transfusion’.

### High burden of deaths following CS

We found that over half of all postnatal maternal deaths followed a CS. This was unexpected given the relatively low national CS rate; estimated at less than 10% of live births.^8 11^ This indicates that women undergoing CS are over-represented among maternal deaths. In comparison with global data, Malawi has a higher proportion of CS-related maternal deaths. Worldwide, 32.8% of postnatal maternal deaths follow a CS; in sub-Saharan Africa this rises to 38%.

The strong association found between CS and maternal death may be attributable to complications caused by the procedure itself. However, CS can also be used as a potentially life-saving intervention for a woman in extremis where death occurs despite the use of the procedure rather than because of it. Most CS in this context are carried out as emergency/unplanned procedures^11 12^ rather than electively, and therefore, carry a higher risk of morbidity and mortality.^8^ A limitation of our analysis is the lack of sufficient data to determine the role of CS in causing the death of the woman, as we did not have information regarding the indication for the procedure. This difficulty has been highlighted by other studies. Sobhy *et al* were unable to adjust for indications for CS in their global meta-analysis of maternal mortality and complications associated with CS in LMICs, nor was this review able to identify whether adverse outcomes observed were due to the procedure itself or from a pre-existing factor. While previous studies have identified obstructed/delayed labours as the most common indication for CS in Malawi^12^ (and in global LMIC settings^8^), further study comparing indications for CS among women who died following the procedure compared with those who survive is required. Furthermore, our data did not include information about whether a CS was carried out as an emergency or a planned procedure. However, it should be noted that CS in the Malawian context is almost exclusively carried out as an emergency/unplanned procedure, and that therefore this can be assumed of the CS carried out among the cohort we report on. From the most recent Demographic and Health Survey (2015–2016), it is known that of the 6% of live births which were delivered by CS in Malawi, only 1% were conducted before the onset of labour pains,^11^

### Causes of death following CS

We found postpartum haemorrhage, pregnancy-specific infection, uterine rupture and antepartum haemorrhage to be important causes of death among women who died following CS. Though we could identify no previous analysis detailing causes of death following CS in Malawi, our findings are in keeping with global data. Sobhy *et al* found that one-third of deaths following CS were attributed to postpartum haemorrhage (32%), one-fifth to sepsis (22%) and one-fifth to pre-eclampsia (19%).^8^ Stratification of cause of death following CS by region or country income level was not included in their analysis for detailed comparison to our findings.

### Avoidable factors

An important feature of our study is the analysis of avoidable health system factors contributing to maternal deaths following CS, allowing deeper insight into the events leading to the woman’s death. Local MDSR committees identified key remediable factors which are important opportunities to prevent avoidable maternal deaths. We found that the factors most frequently associated with death following CS were ‘delay in starting treatment’, ‘inadequate monitoring’, ‘prolonged abnormal observations without action’, ‘lack of essential equipment’ and ‘lack of blood transfusion’. Several factors were significantly more frequently linked to CS births than to vaginal births, including ‘delay in starting treatment’, ‘prolonged abnormal observations without action’, ‘lack of blood transfusion’ and ‘an absence of trained staff on duty’.

Our findings suggest that maternal deaths in this setting result from a complex interaction of human factors and health system constraints including the limited availability of critical resources. Although Malawi has successfully increased the uptake of facility-based birth in recent years, with over 90% of women now delivering at health facilities,^13^ the provision of quality care remains challenging, broadly due unavailability of medications and equipment, substandard infrastructure (eg, electricity, water and transport),^14^ and an unmet need of around 36% in the maternity workforce.^15^ Regarding the context in which CS procedures are carried out, it should be noted that they are generally performed at Central and District hospital level, with some larger primary care facilities also providing this service. Rates of CS are higher in urban centres and among women of the highest educational attainment and wealth quintiles^11^ Outside of the four central hospitals in Malawi, CS are generally carried out by clinical officers, rather than medical doctors. Clinical officers could be better supported by having better access to senior surgical support and enhanced ongoing training and mentorship to develop or improve their surgical skills.

### Strengths and limitations

Our study is strengthened by robust digital data collection from across all government secondary and tertiary level facilities in Malawi. As most women in Malawi deliver at governmental healthcare facilities, and because our data collection was designed to capture the small numbers of death which occurred outside hospital/clinic facilities (around 7%) or at private facilities (13% of births^13^), our sample can be considered representative of the Malawian context. Further strengths include specialist verification of cause of death using an internationally endorsed classification system, novel analysis of deaths following CS in the context of high maternal mortality ratios and the inclusion of an analysis of health system factors linked to maternal deaths.

Limitations of our study include the lack of a surviving group of women with which to compare those women who died following CS. We were only able to include fully audited maternal deaths in our analysis, with the possibility that facilities may have introduced bias in selecting which deaths to audit. Further to this, we were only able to capture deaths which occurred following discharge from hospital if the woman returned to one of the facilities included in the study. Indeed, our estimate of mortality following CS is significantly lower than Sobhy *et al* calculated for sub-Saharan Africa in a meta-analysis of mortality rates following CS (10.9 per 1000 compared with our 3.1 per 1000), though it is in keeping with mortality rates for several individual WHO African Region countries.^8^

### Perspectives for further research

Our analysis indicates that it is necessary to improve the safe and appropriate use of CS in low-resource settings. Interventions to improve maternal health are often developed with vaginal birth in mind, neglecting to benefit those women who give birth surgically. On the other hand, interventions to improve surgical safety are often not relevant or appropriate to obstetric cases. It is, therefore, necessary to develop and trial interventions specific to improving the safety of CS birth in low-resource settings.

From our findings, interventions which may improve outcomes for women undergoing CS could include those aimed at improving general surgical care for obstetric patients, such as the development and implementation of surgical safety checklists specific to CS. They might also include interventions to improve perioperative monitoring of obstetric patients, such as task-shifting the monitoring of vital signs to auxiliary staff, implementation of maternity early warning score systems and standardised guidelines for the care of those recovering following CS. Interventions could also include simulation training, carried out in the theatre setting, to improve skills in managing obstetric emergencies.

Interventions could also be specific to the leading causes of maternal death following CS. For example, postpartum haemorrhage is the leading cause of death among post-CS patients, both in our cohort and globally. A bundled approach to the early detection and management of PPH was recently trialled across several low-resource settings^16^ but focused only on strategies to detect and manage PPH at vaginal birth and did not include women undergoing CS. There remains a need for evidence-based interventions to prevent, detect and managing PPH in CS patients, suitable for use in low-resource settings.

Women who delivered by CS were twice as likely to die from pregnancy-specific infections than other causes of death. Risk factors for infection following CS in low-resource settings include poor infection prevention practices and surgical sterility in the operating theatre,^17^ as well as events before and after surgery.^17^,^21^ As such, further research to determine feasible and effective interventions to improve infection prevention around the time of CS specific to such settings is required. For example, vaginal preparation with antiseptic immediately prior to skin incision to prevent endometritis is well evidenced by studies largely conducted in high-income countries.^22^ However, implementation research to improve the uptake of this intervention in high-need settings would be beneficial.

There is also a need to improve the detection and management of severe infection and sepsis.^23^ For example, through the use of evidence-based maternal sepsis bundles.^23 24^ Furthermore, there exists a paucity of published literature on the microbiology of maternal infection in African low-resource settings to inform international guideline development and clinical management.^25^ Further studies to determine responsible pathogens associated with post-CS infection are, therefore, necessary.

## Conclusion

We conducted an analysis of all postnatal maternal deaths which occurred in Malawi over a 2.5-year period. We found a high burden of maternal deaths following CS, despite a low national CS rate. Over half of all women who died after delivery had undergone this procedure. This proportion is higher than global and regional estimates. We found the leading cause of death following CS to be postpartum haemorrhage, followed by infections and eclampsia. Women who underwent CS were five times more likely to die of infection than those who died following vaginal birth. Multiple remediable health systems factors were identified as contributing to deaths in these women. There is an urgent need to develop and trial interventions to improve the safety of CS in low-resource settings.

## Supplementary material

10.1136/bmjgh-2024-016999 online supplemental file 1

## Data Availability

Data are available on reasonable request.
