# Supplementary material for: Maternal mortality following caesarean section in a low-resource setting: a National Malawian Surveillance Study
Source: BMJ Glob Health. 2024 Nov 24;9(11):e016999. doi: 10.1136/bmjgh-2024-016999 (PMC11590827; doi:10.1136/bmjgh-2024-016999)
Supplement: online supplemental file 1 [file bmjgh-9-11-s001.pdf]

**SUPPLEMENTARY MATERIALS**

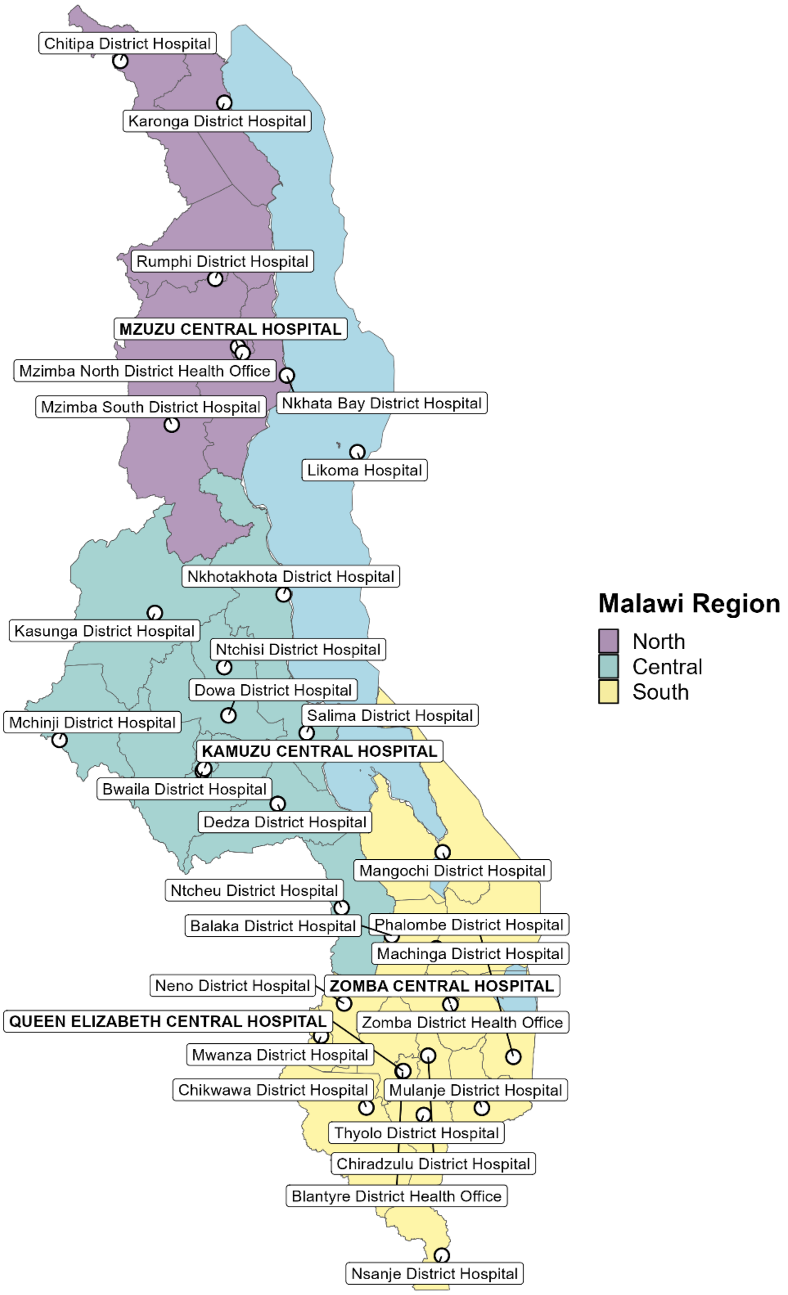


*Figure S1. Map of participating facilities* *(NB Likoma hospital is situated on an island in Lake Malawi)*

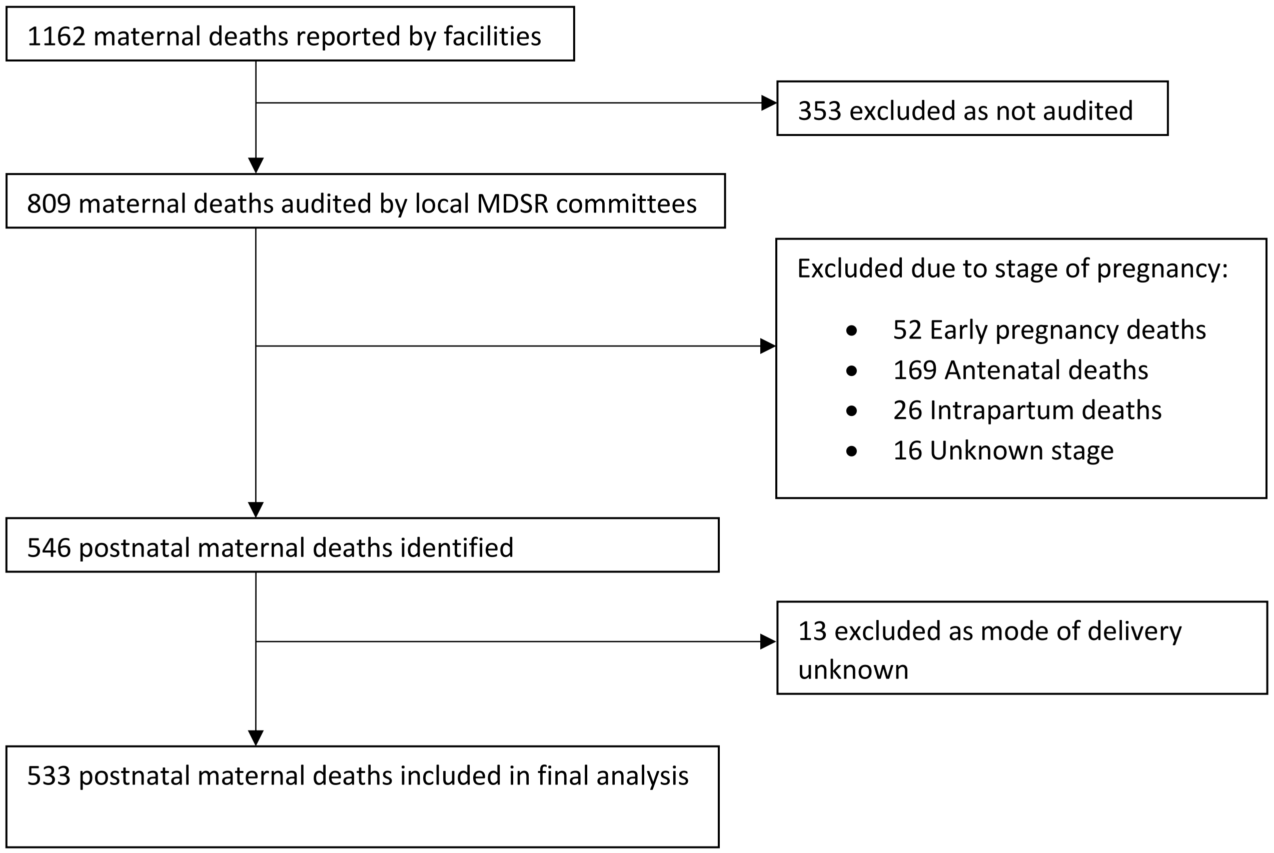


*Figure S2. Study inclusion diagram*

MATSURV MATERNAL HEALTH SURVEILLANCE PLATFORM

http://www.matsurv.org

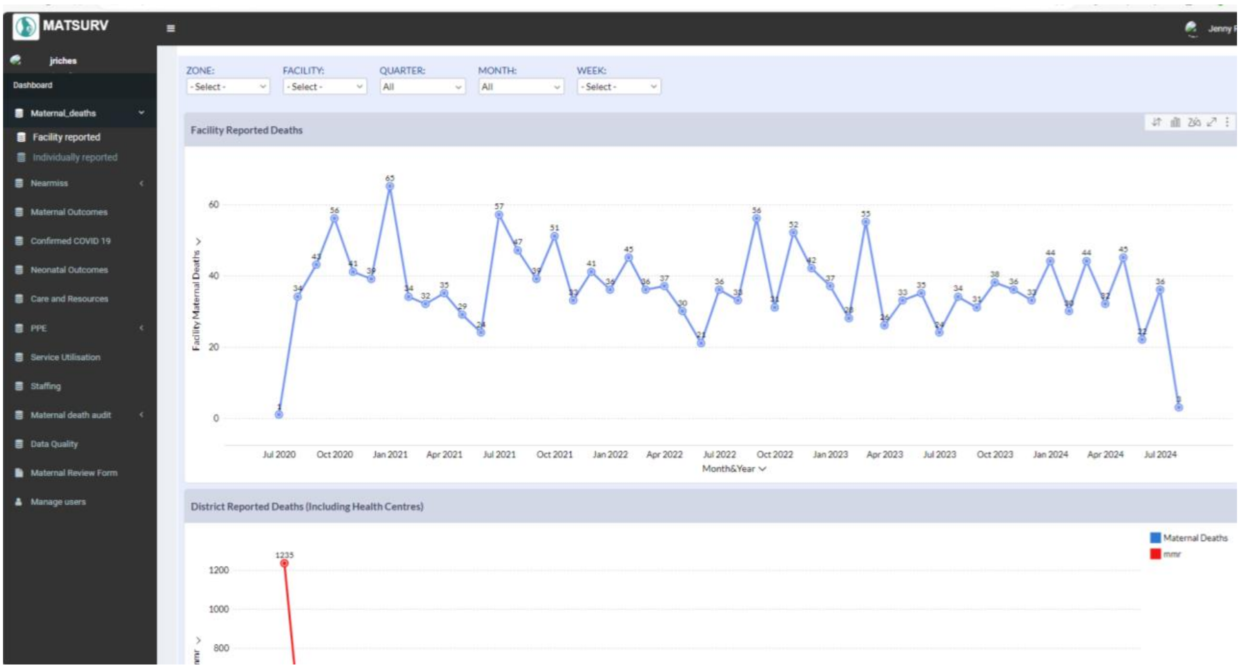


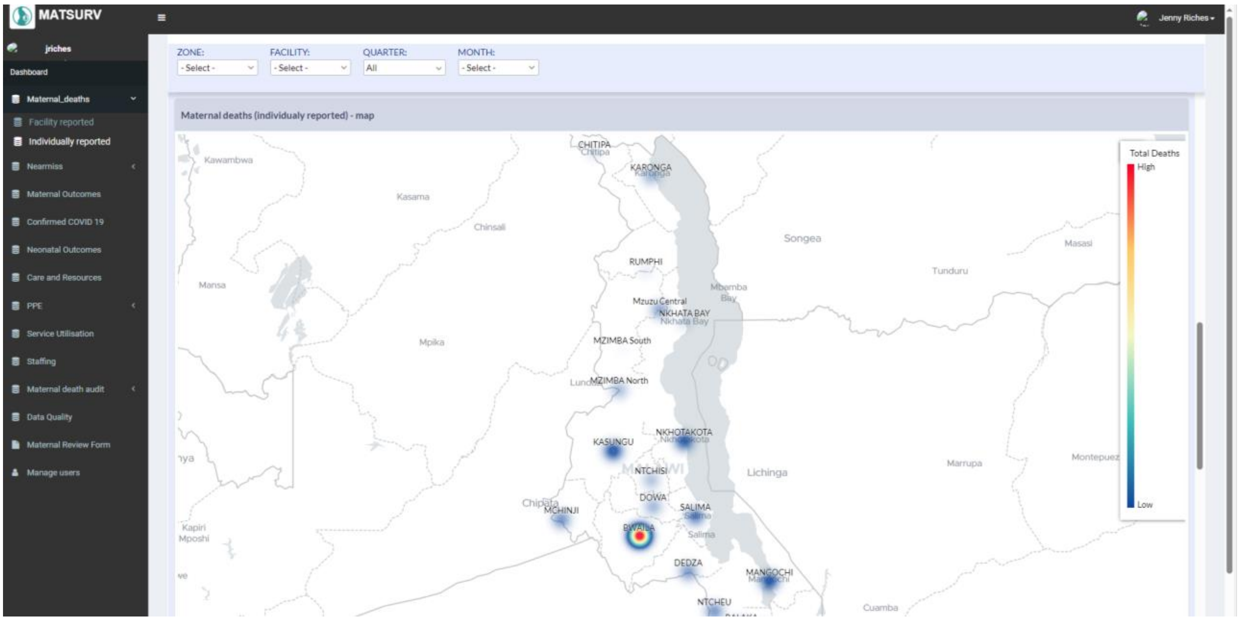


*Figure S3. Matsurv maternal health surveillance platform dashboards*

| Characteristic | Unadjusted Odds Ratio | 95% Confidence Interval | P value |
|---|---|---|---|
| **Age Group** | | | |
| <20 years | - | | |
| 20-25 years | 0.89 | 0.52; 1.52 | 0.666 |
| 26-35 years | 1.1 | 0.67; 1.86 | 0.987 |
| >35 years | 0.99 | 0.57; 1.72 | 0.979 |
| **Marital Status** | | | |
| Married | - | | |
| Single | 0.94 | 0.44; 2.02 | 0.875 |
| Other | 0.94 | 0.27; 3.29 | 0.923 |
| | | | |
| **Education** | | | |
| None | - | | |
| Primary | 1.2 | 0.79; 1.78 | 0.412 |
| Secondary | **2.0** | 1.13; 3.51 | 0.017 |
| Tertiary | **4.1** | 1.45; 11.75 | 0.008 |
| **HIV Status** | | | |
| Negative | - | | |
| Positive | 0.6 | 0.34; 1.08 | 0.089 |
| **HIV on ART** | | | |
| Not taking ART | - | | |
| Taking ART | 0.99 | 0.33; 2.96 | 0.983 |
| **Parity** | | | |
| 0 | - | | |
| 1 | 1.6 | 0.6; 4.08 | 0.364 |
| 2 | 1.2 | 0.43; 3.13 | 0.778 |
| 3 | 1.3 | 0.49; 3.6 | 0.575 |
| >3 | 0.98 | 0.38; 2.52 | 0.968 |
| **Gestation** | | | |
| <28 weeks | - | | |
| 28-31 weeks | 2.5 | 0.69; 9.11 | 0.161 |
| 32-36 weeks | 2.9 | 0.86; 9.52 | 0.088 |
| 37-42 weeks | 3.1 | 0.98; 10.06 | 0.054 |
| **ANC attendance** | | | |
| No ANC | - | | |
| Received ANC | **3.5** | 1.54; 7.97 | 0.002 |
| **Location admitted from** | | | |
| Another facility | - | | |
| Home/Community | 0.92 | 0.65; 1.29 | 0.617 |
| **Condition at admission** | | | |
| Stable | - | | |
| Critically ill | **0.51** | 0.36; 0.72 | <0.001 |
| Dead on arrival | **0.085** | 0.02; 0.29 | <0.001 |

*Table S1. Unadjusted odds ratios for maternal death following CS by demographic and clinical exposures*

| Health system factors | Vaginal birth (n=257) | | CS (n=276) | | P value |
|---|---|---|---|---|---|
| | n | (Proportion %) | n | (Proportion %) | |
| **Healthcare worker factors** | | | | | |
| Any healthcare worker factor | 221 (86.0%) | | 246 (89.1%) | | 0.272 |
| Inadequate midwifery skills | 60 (23.3%) | | 57 (20.7%) | | 0.453 |
| Uncertified provider | 2 (0.8%) | | 2 (0.7%) | | 0.943 |
| Delay in deciding to refer | 49 (19.1%) | | 66 (23.9%) | | 0.174 |
| Initial assessment incomplete | 100 (38.9%) | | 96 (34.8%) | | 0.323 |
| Inadequate resuscitation | 116 (45.1%) | | 106 (38.4%) | | 0.115 |
| **Wrong diagnosis** | **33 (12.8%)** | | **21 (7.6%)** | | **0.045** |
| Partograph incorrectly/not used | 33 (12.8%) | | 28 (10.1%) | | 0.329 |
| Wrong treatment | 30 (11.7%) | | 29 (10.5%) | | 0.668 |
| Unsafe medical treatment | 8 (3.1%) | | 16 (5.8%) | | 0.135 |
| No treatment | 12 (4.7%) | | 15 (5.4%) | | 0.687 |
| **Delay in starting treatment** | **90 (35.0%)** | | **129 (46.7%)** | | **0.006** |
| Inadequate monitoring | 103 (40.1%) | | 128 (46.4%) | | 0.143 |
| **Prolonged abnormal observations without action** | **77 (30.0%)** | | **114 (41.3%)** | | **0.006** |
| Lack of obstetric lifesaving skills | 34 (13.2%) | | 46 (16.7%) | | 0.267 |
| **Administrative factors** | | | | | |
| Any administrative factor | 130 (50.6%) | | 141 (51.1%) | | 0.908 |
| Communication problems between facilities | 17 (6.6%) | | 22 (8.0%) | | 0.548 |
| **Transport problems between facilities** | **25 (9.7%)** | | **10 (3.6%)** | | **0.004** |
| Lack of qualified staff | 5 (1.9%) | | 7 (2.5%) | | 0.646 |
| Lack of antibiotics | 7 (2.7%) | | 5 (1.8%) | | 0.478 |
| Lack of essential obstetric drugs | 12 (4.7%) | | 18 (6.5%) | | 0.354 |
| Lack of essential equipment | 60 (23.3%) | | 69 (25.0%) | | 0.656 |

| | | | |
|---|---|---|---|
| Lack of laboratory facilities | 27 (10.5%) | 24 (8.7%) | 0.478 |
| **Lack of blood transfusion** | **24 (9.3%)** | **43 (15.6%)** | **0.030** |
| **Absence of trained staff on duty** | **2 (0.8%)** | **11 (4.0%)** | **0.016** |
| **Patient or Family factors** | | | |
| **Any patient or family associated factors** | **142 (55.3%)** | **100 (36.2%)** | **<0.001** |
| **Delay in reporting to the health facility** | **115 (44.7%)** | **68 (24.6%)** | **<0.001** |
| Lack of transport from home to facility | 7 (2.7%) | 3 (1.1%) | 0.164 |
| Unsafe traditional/cultural practices | 8 (3.1%) | 5 (1.8%) | 0.330 |
| Unsafe self-medication | 4 (1.6%) | 3 (1.1%) | 0.634 |
| Refusal of treatment | 13 (5.1%) | 10 (3.6%) | 0.415 |
| **Delay in decision-making** | **88 (34.2%)** | **48 (17.4%)** | **<0.001** |
| Use of traditional medicine/practices | 6 (2.3%) | 8 (2.9%) | 0.684 |
| **TBA/Community factors** | | | |
| **Any traditional birth attendant or Community factor** | **27 (10.5%)** | **16 (5.8%)** | **0.046** |
| Failure to recognise danger signs | 19 (7.4%) | 10 (3.6%) | 0.055 |
| Failure to accept limitations | 10 (3.9%) | 7 (2.5%) | 0.374 |
| Use of traditional medicine | 3 (1.2%) | 0 (0.0%) | 0.072 |
| Lack of transport | 1 (0.4%) | 1 (0.4%) | 0.060 |
| Delay in deciding to refer | 12 (4.7%) | 6 (2.2%) | 0.111 |

*Table S2. Health system factors associated with maternal deaths by mode of delivery*

| Cause of death | CS (n=262) | | Vaginal birth (n=242) | |
|---|---|---|---|---|
| | Proportion (95% CI) | | Proportion (95% CI) | |
| **Postpartum haemorrhage** | 68 | 26.0% (20.8-31.8%) | 93 | 38.4% (32.3-44.9%) |
| **Eclampsia** | 41 | 15.6% (11.6-20.7%) | 28 | 11.6% (8.0-16.4%) |
| **Pregnancy-specific infection[1]** | 37 | 14.1% (10.3-19.1%) | 28 | 11.6% (8.0-16.4%) |
| **Ruptured Uterus** | 31 | 11.8% (8.3-16.5%) | 9 | 3.7% (1.8-7.1%) |
| **Antepartum haemorrhage** | 26 | 9.9% (6.7-14.4%) | 1 | 0.4% (0.02-2.6%) |
| **Pre-eclampsia** | 22 | 8.3% (5.5-12.6%) | 19 | 7.8% (4.9-12.2%) |
| **Non-pregnancy specific infection[2]** | 15 | 5.7% (3.4-9.5%) | 42 | 17.4% (12.9-22.9%) |
| **Complications of anaesthesia** | 8 | 3.1% (1.4-6.2%) | 0 | - |
| **Venous complications in pregnancy** | 5 | 1.9% (0.7-4.6%) | 2 | 0.8% (0.1-3.3%) |
| **Peripartum cardiomyopathy** | 5 | 1.9% (0.7-4.6%) | 4 | 1.7% (0.5-4.5%) |
| **Abortion/Miscarriage** | 0 | - | 1 | 0.4% (0.02-2.6%) |
| **Diabetes** | 0 | - | 1 | 0.4% (0.02-2.6%) |
| **Obstetric embolism** | 1 | 0.4% (0.02-2.4%) | 0 | - |
| **Cardiovascular disease** | 0 | - | 4 | 1.7% (0.5-4.5%) |
| **Gastrointestinal conditions** | 0 | - | 3 | 1.2% (0.3-3.9%) |
| **Central nervous system conditions** | 0 | - | 1 | 0.4% (0.02-2.6%) |
| **Respiratory conditions** | 2 | 0.8% (0.1-3.0%) | 3 | 1.2% (0.3-3.9%) |
| **Cancer** | 0 | - | 1 | 0.4% (0.02-2.6%) |
| **Anaemia** | 0 | - | 2 | 0.8% (0.1-3.3%) |
| **Haematological** | 1 | 0.4% (0.02-2.4%) | 0 | - |

*Table S3. Causes of postnatal maternal death by mode of delivery*

[1]Pregnancy-specific infection refers to infections directly related to pregnancy such as chorioamnionitis or endometritis.
[2]Non-pregnancy specific infection refers to infections not directly related to pregnancy such as malaria or tuberculosis

| **Cause of death** | **Unadjusted Odds Ratio** | **95% Confidence Interval** | **P value** |
|---|---|---|---|
| Postpartum haemorrhage | **0.59** | 0.41; 0.85 | 0.005 |
| Eclampsia | 1.4 | 0.84; 2.3 | 0.201 |
| Pregnancy-specific infection | 1.2 | 0.74; 2.06 | 0.424 |
| Ruptured Uterus | **3.0** | 1.46; 6.03 | 0.003 |
| Antepartum haemorrhage | **14** | 3.26; 59.1 | <0.001 |
| Pre-eclampsia | 1.1 | 0.6; 2.16 | 0.688 |
| Non-pregnancy specific infection | **0.3** | 0.16; 0.56 | <0.001 |

*Table S4. Unadjusted odds ratios for death following CS for each leading cause of maternal death**

*Causes of death which contributed less than 5% to the total number of postnatal maternal deaths have been excluded from this table.
